# Urinary tract infection in diabetics hospitalized in Befelatanana Hospital, Antananarivo: Epidemiological, clinical, biological profiles and risk factors for multidrug‐resistant bacterial infection

**DOI:** 10.1002/ccr3.7867

**Published:** 2023-09-04

**Authors:** Rija Eric Raherison, Sitraka Angelo Raharinavalona, Thierry Razanamparany, Theo Njakasoa Randrianotahiana, Tsikinirina Valisoa Randrianomanana, Miora Maeva Arielle Andrianiaina, Andrianirina Dave Patrick Rakotomalala, Radonirina Lazasoa Andrianasolo

**Affiliations:** ^1^ Endocrinology Department Joseph Raseta Befelatanana University Hospital Center Antananarivo Madagascar; ^2^ Internal Medicine and Cardiovascular Diseases Departments Soavinandriana Hospital Center Antananarivo Madagascar

**Keywords:** antibiotics, diabetes mellitus, *Escherichia coli*, pyelonephritis, sensitive

## Abstract

**Key Clinical Message:**

The main type of urinary tract infection in hospitalized diabetics in Antananarivo is acute pyelonephritis; *Escherichia coli* is the most isolated uropathogen; imipenem, amikacin, fosfomycin and ceftriaxone are the major antibiotics for which *Escherichia coli* retain good sensitivity; Type 2 diabetes is predictive factor for infection by multidrug resistant bacteria.

**Abstract:**

This study aimed to describe the epidemiological‐clinical profiles of diabetics hospitalized for bacterial urinary tract infections in the Endocrinology Department of Befelatanana Hospital, to identify the main bacteria responsible, their antibiotic sensitivity profile and the factors associated with multidrug‐resistant bacterial infection. A cross‐sectional study was conducted between March 2017 and March 2020 involving all diabetics hospitalized for documented community‐acquired bacterial urinary tract infection during this period. The hospital prevalence of urinary tract infections was 4.64%. The mean age of the patients was 59.06 ± 14.26 years and the sex ratio was 0.15. The main sign was fever (55.76%). The main clinical form was uncomplicated acute pyelonephritis (38.46%). Fifty‐seven bacterial uropathogens were isolated. The most frequent was *Escherichia coli* (77.19%). *Escherichia coli* was sensitive to ertapenem and nitrofurantoin in 100% of cases, to Amikacin in 97.5% of cases, to Fosfomycin in 94.4% of cases and to Ceftriaxone in 80.65% of cases. Thirteen patients were infected with multidrug‐resistant bacteria, all of them are extended‐spectrum beta‐lactamase‐producing Enterobacteriaceae. Only the type of diabetes was associated with multidrug‐resistant bacteria infection. The epidemiological‐clinical and biological characteristics of urinary tract infections in our diabetics are similar to those reported in the literature. Compliance with the rules of proper antibiotic use is imperative to limit the emergence and spread of multidrug‐resistant bacteria.

## Graphical abstract

We found that the patients were predominantly female, the average age was around 60, severe renal failure was predominant as a risk factor for urinary tract infection complications, diabetes was often poorly controlled, and *Escherichia coli* was predominant as a bacterial uropathogen. Their sensitivities to carbapenems and amikacin are excellent. Ceftriaxone and ciprofloxacin, which are easily accessible molecules, may still have a place in the treatment of parenchymal urinary tract infections, provided that the rules of proper antibiotic use are followed.

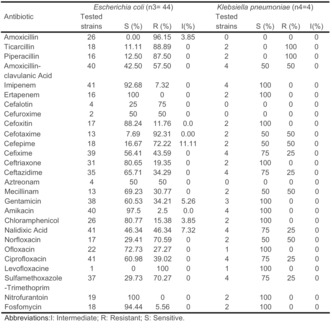

## 1 BACKGROUND

Urinary tract infection (UTI) is an infection of any part of the urinary system (kidneys, ureters, bladder, and urethra) by one or more microorganisms.^1^ Diabetic subjects have an increased risk of UTI due to glycosuria, immunodeficiency and neurological and vascular complications caused by the disease, its impact on the cellular barrier and local defenses altering the activity of polynuclears and phagocytosis.^2^, ^3^ In Antananarivo, UTI occupied the third place among community infections among diabetics hospitalized at the Endocrinology Department of Befelatanana Hospital in 2015.^4^

In the literature, *Escherichia coli* remain the main bacterial uropathogens responsible for UTI in both diabetics and nondiabetics.^5^, ^6^ The high frequency of UTI has led to a high rate of antibiotic prescription.^7^, ^8^ This may contribute to the spread of multidrug‐resistant bacteria (MDR), which is a major public health problem.^9^ As with any infection, knowledge of the resistance profile is necessary to guide prescribing. Indeed, it has also been reported that the appropriate use of antibiotics in patients with complicated UTI appears to reduce the length of hospital stay and thus has a favorable effect on health care outcomes and costs.^10^

To our knowledge, few studies have been done to UTI in Malagasy diabetics, particularly concerning their bacteriological profile and their antibiotic sensitivity. Moreover, knowledge of the factors associated with UTI with MDR will be of great use, as it will inevitably have an impact on the choice of antibiotics, the strategy and the preventive measures to be implemented in order to limit the spread of MDR.

The aims of this study were to describe the epidemiological‐clinical profiles of diabetic patients hospitalized for UTI, to identify the main bacterial uropathogens responsible for these UTI, their antibiotic sensitivity profile and the factors associated with UTI due to a MDR. This in order to facilitate and make early the diagnosis of urinary tract infection on the one hand and on the other hand to propose empirical antibiotic therapy adapted to the local context where bacteriological examinations are often lacking.

This study was carried out at the Endocrinology Department of Befelatanana Hospital. This department is the national reference service for diabetology. As it is a reference center, although the hospital is located in the city center of Antananarivo, the capital of Madagascar, patients come from several regions of Madagascar.

Thus, the characteristics of the patients admitted there reflect, in a way, those of the other regions.

## 2 MATERIALS AND METHODS

### 2.1 Study design and patients

A cross‐sectional study was conducted including all previously known or newly diagnosed diabetic patients hospitalized in this department from March 1, 2017 to March 31, 2020 (37 months).

All those with a bacterial community‐acquired UTI confirmed by urine culture according to the bacteriological definition of an UTI (leukocyturia ≥104/mL, bacteriuria ≥103/mL or 104/mL depending on the bacterial uropathogens and the gender)^3^ and who had benefited from antibiotic sensitivity testing were included. Pregnant women, those whose results were in favor of urinary contamination characterized by the presence of urinary epithelial cells and/or a culture finding more than two types of colonies and patients discharged against medical advice were excluded from the study.

Clinical forms were selected according to the criteria from the recommendations of the French Infectious Diseases Society (SPILF) in 2017.^3^ The diagnosis of diabetes mellitus is confirmed by the criteria of the American Diabetes.

Association (ADA).^11^

### 2.2 Clinical and laboratory data

Epidemiological‐clinical and biological variables were analyzed.

Epidemiological data included gender, age, and characteristics of diabetes including type, duration, and known degenerative complications.

The clinical data included UIT's complication risk factors, the UTI's clinical signs, the duration of UTI's clinical sign before hospitalization, the notion of taking antibiotics before hospitalization and the type of UTI.

Biological variables include the admission blood glucose, glycated hemoglobin level, and results of the urine culture and antibiotic sensitivity test.

A single urine sample was taken on admission for each patient. The sample collection was preceded by prior cleansing of the external genital tract with a non‐foaming antiseptic pad, followed by rinsing with saline. The urine collected was that of the “midstream” for non‐catheterized patients. For patients with a bladder catheter, the catheter was disinfected with an antiseptic before being punctured with a needle to collect the urine for analysis. The urine was quickly transported to the laboratory and was examined by direct microscopy, Gram staining, uroculture and biochemical and metabolic characterization of the bacterial uropathogen found.

Antibiogram was performed by disk diffusion method to determine the antibiotic sensitivity of isolates. The result was interpreted as sensitive, intermediate and resistant. Multidrug‐resistance is defined as non‐sensitivity to at least one antibiotic in three or more antibiotic categories.^12^

### 2.3 Statistical analysis

The data were collected from a preestablished collection grid, from patient files. They were used by Epi Info™ software version 3.5.4. (United States Centers for Disease Control and Prevention in Atlanta, Georgia). The qualitative and quantitative variables are expressed respectively as a percentage and as an average with standard deviation.

After a descriptive study, a univariate statistical analysis was performed according to the presence of MDR using the odds ratio with 95% confidence interval and statistical significance *p* < 0.05.

Patients' anonymity was preserved by a coding method. Confidentiality was respected.

This study was approved by the Befelatanana Hospital manager (cf. Hospital manager approbation letter on September 2, 2022).

## 3 RESULTS

Among the 1119 diabetics hospitalized during the study period, 66 patients had symptoms suggestive of UTI and benefited a urine culture. Fourteen patients were excluded because for 10 of them, the urine culture was in favor of urinary contamination and four patients were discharged against medical advice. In the end, 52 patients were included in the study, giving a hospital incidence of UTI of 4.64%.

Table 1 summarized the epidemiological and diabetes characteristics of our patients. The mean age of our patients was 59.06 ± 14.26 years(18–83 years). Of these, 45 (86.54%) were female. Sex ratio was 0.15. Type 2 diabetics were predominant at 78.84%. For eight patients (15.38%), diabetes was discovered during hospitalization. The average duration of diabetes for subjects already known to be diabetic before hospitalization was 7.41 ± 7.72 years (0.17–36 years).The main known chronic complications of diabetes at admission were nephropathy (26.92%)and stroke (7.69%).

**TABLE 1:** Epidemiological and diabetes characteristics of patients (*N* = 52).

Variables	Number (*N* = 52)	Proportion (%)
Gender		
Male	7	13.46
Female	45	86.54
Age (years old)		
18–20	1	1.92
21–30	1	1.92
31–40	4	7.69
41–50	6	11.54
51–60	11	21.15
61–70	17	32.69
71–80	11	21.15
81–90	1	192
Type of diabetes		
Type 1	11	21.15
Type 2	41	78.85
Diabetes duration (years)		
Newly diagnosed diabetes	8	15.38
<1	4	7.69
1–5	22	42.31
6–10	8	15.38
>10	10	19.23
Degenerative complication of diabetes		
Nephropathy	14	26.92
Stroke	4	7.69
Cardiopathy	3	5.77
Diabetic foot	2	3.85
Peripheral neuropathy	1	1.92
Autonomic neuropathy	1	1.92
Retinopathy	1	1.92
Peripheral arterial disease	1	1.92

The clinical characteristics of UTI are summarized in Table 2. Severe chronic renal failure was the leading risk factor for UTI complications (23.08%), followed by uropathy (21.15%). A patient could have one or more risk factors.

**TABLE 2:** Clinical characteristics of patients (*N* = 52).

Variables	Number (*N* = 52)	Proportion (%)
UTI complication risk factors		
Severe chronic renal failure	12	23.08
Uropathy	11	21.15
Male gender	7	13.46
Age > 75 years old	6	11.54
History of UTI	3	5.77
Age > 65 years old + ≥ 3 frailty criteria	2	3.85
Immunodeficiency	1	1.92
Clinical signs		
Fever	29	55.76
Flank/loin pain	14	26.92
Burning with urination	14	26.92
Abdominal pain	13	25
Dysuria	10	19.23
Deterioration of general condition	8	15.38
Diarrhea	8	15.38
Thrill	6	11.54
Pollakiuria	6	11.54
Acute urinary retention	4	7.69
Urge urination	1	1.92
Duration of UTI's clinical signs (day)		
1–5	26	50
6–10	17	32.69
11–15	5	9.62
16–20	0	0
21–25	1	1.92
26–30	2	3.85
>30	1	1.92
Antibiotic using before hospitalization		
Yes	5	9.61
No	47	90.38
Types of UTI		
Uncomplicated APN	20	38.46
APN at risk of complications	13	25
Prostatitis	7	13.46
Uncomplicated cystitis	5	9.62
Severe APN	5	9.62
Cystitis at risk of complication	2	3.85

Abbreviations: APN, acute pyelonephritis; UTI, urinary tract infection.

Fever was the most reported clinical sign (29 patients i.e., 55.76%), followed by flank/loin pain and burning with urination (14 patients i.e., 26.92% respectively). A patient could present one or more signs at admission. Clinical signs had been evolving, on average, for 7.90 ± 9.84 days (1–60 days).Five patients (9.61%) had already taken antibiotics before admission. Uncomplicated acute pyelonephritis (ANP) was predominant with 20 cases out of 52, that is, 38.46%, followed by ANP at risk of complications (13 cases out of 52, i.e., 25%).

The paraclinical characteristics of UTI are grouped in Table 3. The mean blood glucose level on admission was 290 ± 152 mg/dL (20–600 mg/dL) [16.095 ± 8.43 mmol/L (1.11–33.3 mmol/L)]. Only 24 patients had a glycated hemoglobin (HbA1c) measurement during hospitalization with a mean level of 9.40 ± 2.93% (4.17–15.4%) [79 ± 9 mmol/mol (22–145 mmol/mol)]. Fifty‐seven bacterial uropathogens were isolated from the 52 patients, with an average of 1.09 bacterial isolates per patient. For 4 patients (7.69%), urine culture found 2 different isolates. For the rest, the infection was monomicrobial. *Escherichia coli* (77.19%), *Staphylococcus aureus* (8.77%) and *Klebsiella pneumoniae* (7.02%) were the predominant bacterial uropathogens identified in terms of frequency.

**TABLE 3:** Paraclinical characteristics of patients (*N* = 52).

Variables	Number (*N* = 52)	Proportion (%)
Admission blood glucose (mg/dL)		
<80	3	5.77
80–130	4	7.69
130–180	8	15.38
180–200	2	3.85
200–300	11	21.15
300–400	12	23.08
400–500	6	11.54
>500	6	11.54
Glycated hemoglobin (%)	(*n*1 = 24)	
<6.5%	2	8.33
6.5–7	3	12.50
7–8	6	25
8–9	4	16.67
>9	9	37.50
Bacterial uropathogen	(*n*2 = 57)	
*Escherichia coli*	44	77.19
*Staphylococcus aureus*	5	8.77
*Klebsiella pneumoniae*	4	7.02
*Proteus mirabilis*	2	3.51
*Enterococcus* sp.	1	1.75
*Klebsiella oxytoca*	1	1.75

Regarding the results of the antibiogram, for the two main Enterobacteriaceae that is, *Escherichia coli* and *Klebsiella pneumoniae*, their antibiotic sensitivity are summarized in Table 4. In decreasing order of frequency, antibiotic for which *Escherichia col*i was most sensitive were ertapenem (100%), nitrofurantoin (100%), amikacin (97.5%), fosfomycin (94.4%) and ceftriaxone (80.65%). On the contrary, their resistance to more conventional antibiotic was high. Respectively, *Escherichia coli* was resistant in 96.15% of cases to Amoxicillin, in 70.27% of cases to sulfamethoxazole‐trimethoprim, in 57.5% of cases with Amoxicillin‐Clavulanic Acid, in 34.21% of cases with Gentamicin. *Klebsiella pneumoniae* was sensitive in 100% of cases to: imipenem, ertapenem, cefoxitin, ceftriaxone, gentamicin, amikacin, chloramphenicol, ofloxacin, fosfomycin, nitrofurantoin and in 75% of cases each to cefixime, ciprofloxacin and Sulfamethoxazole‐Trimethoprim.

**TABLE 4:** Results of antibiogram for *Escherichia coli* and *Klebsiella pneumoniae.*

Antibiotic	*Escherichia coli* (*n*3 = 44)				*Klebsiella pneumoniae* (n4 = 4)			
	Tested strains	Sensitive (%)	Resistant (%)	Intermediate (%)	Tested strains	Sensitive (%)	Resistant (%)	Intermediate (%)
Amoxicillin	26	0.00	96.15	3.85	0	0	0	0
Ticarcillin	18	11.11	88.89	0	2	0	100	0
Piperacillin	16	12.50	87.50	0	2	0	100	0
Amoxicillin‐ clavulanic acid	40	42.50	57.50	0	4	50	50	0
Imipenem	41	92.68	7.32	0	4	100	0	0
Ertapenem	16	100	0	0	2	100	0	0
Cefalotin	4	25	75	0	0	0	0	0
Cefuroxime	2	50	50	0	0	0	0	0
Cefoxitin	17	88.24	11.76	0.0	2	100	0	0
Cefotaxime	13	7.69	92.31	0.00	2	50	50	0
Cefepime	18	16.67	72.22	11.11	2	50	50	0
Cefixime	39	56.41	43.59	0	4	75	25	0
Ceftriaxone	31	80.65	19.35	0	2	100	0	0
Ceftazidime	35	65.71	34.29	0	4	75	25	0
Aztreonam	4	50	50	0	0	0	0	0
Mecillinam	13	69.23	30.77	0	2	50	50	0
Gentamicin	38	60.53	34.21	5.26	3	100	0	0
Amikacin	40	97.5	2.5	0.0	4	100	0	0
Chloramphenicol	26	80.77	15.38	3.85	2	100	0	0
Nalidixic Acid	41	46.34	46.34	7.32	4	75	25	0
Norfloxacin	17	29.41	70.59	0	2	50	50	0
Ofloxacin	22	72.73	27.27	0	1	100	0	0
Ciprofloxacin	41	60.98	39.02	0	4	75	25	0
Levofloxacine	1	0	100	0	1	100	0	0
Sulfamethoxazole‐Trimethoprim	37	29.73	70.27	0	4	75	25	0
Nitrofurantoin	19	100	0	0	2	100	0	0
Fosfomycin	18	94.44	5.56	0	2	100	0	0

For the main Gram‐positive cocci (GPC) isolated that is, *Staphylococcus aureus*, the result of the antibiogram was represented in Table 5. All strains were sensitive to oxacillin, gentamicin, erythromycin, ciprofloxacin and sulfamethoxazole‐trimethoprim. Regarding the general antibiotic resistance profile of all bacterial uropathogens, thirteen urinary culture (22.80%) had isolated MDR, all extended‐spectrum beta lactamase‐producing Enterobacteriaceae (ESBLE). Twelve of the 44 isolated *Escherichia coli* (27.27%) and 1 of the 4 isolated *Klebsiella pneumoniae* (25%) were ESBLE.

**TABLE 5:** Results of antibiogram for *Staphylococcus aureus* (*n*5 = 5).

Antibiotic	*Staphylococcus aureus* (*n*5 = 5)			
	Tested strains	Sensitive (%)	Resistant (%)	Intermediate (%)
Oxacillin	5	100	0	0
Penicillin G	5	0	100	0
Amoxicillin	0	0	0	0
Cefoxitin	2	100	0	0
Gentamicin	3	100	0	0
Kanamycin	2	100	0	0
Tobramycin	2	100	0	0
Erythromycin	3	100	0	0
Lincomycin	3	100	0	0
Clindamycin	2	100	0	0
Vancomycin	1	100	0	0
Fusidique acid	2	100	0	0
Rifampicin	2	100	0	0
Norfloxacin	3	100	0	0
Ciprofloxacin	2	100	0	0
Levofloxacin	0	0	0	0
Sulfamethoxazole‐trimethoprim	3	100	0	0
Nitrofurantoin	2	100	0	0

Only the type of diabetes was associated with multidrug‐resistant bacteria infection (Table 6).

**TABLE 6:** Factors associated with urinary tract infection by multidrug resistant bacterial.

Variables	MDR (*n*6 = 13)	No MDR (*n*7 = 39)	OR [95% CI]	*p*‐Value
Male gender	1 (14.28%)	6 (85.71%)	2.25 [0.2447–20.6869]	0.41
Age ≥ 65 years old	4 (22.22%)	14 (77.77%)	1.31 [0.3404–5.0614]	0.48
Type 1 diabetes	0 (0%)	10 (100%)		0.0036
Type 2 diabetes	13 (30.95%)	29 (69.04%)		
Antibiotic using before hospitalization	2 (40%)	3 (60%)	2.1212 [0.3131–14.3706]	0.37
Uncomplicated APN	5 (25%)	15 (75%)	0.9583 [0.263–3.4916]	0.60
APN at risk of complication	4 (30.76%)	9 (69.23%)	1.6667 [0.4058–6.8445]	0.35
Prostatitis	1 (14.28%)	6 (85.71%)	0.4444 [0.0483–4.0863]	0.41
Uncomplicated cystitis	1 (20%)	4 (80%)	0.7083 [0.0719–6.981]	0.62
Severe APN	2 (40%)	3 (60%)	2.1212 [0.3131–14.3706]	0.37
Cystitis at risk of complication	0 (0%)	2 (100%)	–	0.55
Admission blood glucose ≥180 mg/dL	7 (35%)	13 (65%)	0.3621 [0.0965–1.3584]	0.11
HbA1c ≥ 7%	7 (36.84%)	12 (63.15%)	2.3333 [0.2157–25.2461]	0.44

Abbreviations: APN, acute pyelonephritis; CI, confidence interval; MDR, Multidrug‐resistant bacterial; OR, odds ratio.

## 4 DISCUSSION

The prevalence of UTI found in this study (4.64%) was similar to that reported by Norafika et al. in Indonesia (3.93%)^13^ but differed from those reported by other authors including Yenehun Worku et al. in Ethiopia (9.8%)^14^ and Raherison et al. in Madagascar (18.52%).^4^ The variability of the frequency of UTI reported in diabetics can be likely explained by the difference in the study methodologies used. Indeed, like us, Norafika et al. had performed a cross‐sectional study including hospitalized diabetics with a bacteriologically documented UTI,^13^ which explains the similarity of our results. On the other hand, for. Yenehun Worku et al., the study was carried out on all diabetic patients followed up at the Zewditu Memorial Hospital in Addis Ababa, whether they were symptomatic or not.^14^ For Raherison et al., although the study was performed at the same site as ours, the frequency of UTI found was higher than ours since their diagnosis of UTI was based mainly on clinical criteria and the presence of leukocyturia and nitrite on urine test strip examination.^4^

The average age of our patients (59.06 years) was similar to that of Affes et al. (57 years).^15^ Indeed, urinary stasis favored by prostatic hyperplasia, possible at this age in men, and the colonization of urine by uropathogenic strains linked to the modification of vaginal flora in postmenopausal women could explain this age of predilection for UTI. Also, as the majority of our patients suffered from Type 2 diabetes, it is normal that our patients were close to 60 years old on average. The female predominance of UTI is also widely reported by another authors.^16^, ^17^ It could be explained by the short length of the female urethra and the proximity of the urethral meatus to the vagina and anus, which favors the occurrence of UTI.^18^

In our country, infections are still one of the main complications of diabetes mellitus. The average duration of diabetes in our patients already known to be diabetic before hospitalization (7.41 years) close to that of other authors such as Mariko et al (7.45 years)^19^ and Shah et al (8.06 ± 7.07 years).^17^ This duration corresponds approximately to the average time for the occurrence of degenerative complications, particularly microvascular ones, which would favor the occurrence of infections.^20^

The main complication of diabetes reported by our patients was diabetic nephropathy (28.57%), similarly to that reported by Bagir et al. (29.3%).^16^

In this study, 25% of our patients had severe chronic renal failure. This is in line with the result reported by Ahmad's study (38.05%).^21^ It is known that renal failure is one of the risk factors of immunodeficiency and complication of UTI,^3^ which also requires the adaptation of antibiotic dose in order to avoid major adverse effects due to the lack of its elimination.^22^ The other main complication of diabetes was stroke. Indeed, neurological sequel such as autonomic neuropathy and bed rest would favor the occurrence of UTI due to urinary stasis. Like Gninkoun et al.,^23^ we found that the main signs of UTI were fever, flank/loin pain and burning with urination.

One patient out of 10 had already taken an antibiotic before hospitalization. However, we were not able to know whether or not these antibiotics were prescribed by health professionals and according to the rules of good antibiotic use. Moreover, in Madagascar, self‐medication and the illicit sale of drugs, including antibiotics, are still common.

In this study, uncomplicated ANP was the most common form of UTI. This was in contradiction to the result of the study conducted by Shah et al.^17^ which found 43.6% of cystitis and 7.9% of ANP and that of Gninkoun et al.^23^ which reported 76.08% of cystitis and 6.52% of ANP. The difference might be due, in part, to the fact that we only took into account UTI leading to hospitalization, whereas for these other authors, it was not specified whether UTI was the main diagnosis. However, it is known that diabetic subjects suffer 4 to 5 times more pyelonephritis than nondiabetics^24^ and diabetes increases the risk of hospitalization for pyelonephritis by a factor of 3.^25^ Cystitis, which is less severe and does not present systemic symptoms, can be treated on an outpatient basis, which explains why it is less frequent in hospitalization.

Most of our patients had poorly controlled diabetes before the UTI, based on the mean HbA1c of those who had benefited from its dosage (9,40%). A similar situation was also found by the Bagir et al. (9.3%).^16^ Indeed, the poor glycemic control would be at the origin of the presence of glycosuria favoring urinary microbial overgrowth, of a failure of the autonomic nervous system altering the dynamics of the excretory tract, and of the imbalance of the immune system disturbing the immune response.^26^ Furthermore, the blood glucose level at admission of our patients was similar to that reported by Bagir et al. (220 ± 107 mg/dL).^16^ This could be explained by the fact that the diabetes was already poorly controlled and also that infection, like any state of stress, raises blood glucose levels.^27^

Enterobacteriaceae predominated in our study, including *Escherichia coli* (77.19%) and *Klebsiella pneumoniae* (7.02%). Among the GPC, *Staphylococcus aureus* was predominant (8.77%). These main bacterial uropathogens were also found by other authors such as Zubair et al.^28^ in Pakistan (71% for *Escherichia coli*, 7.48% for *Klebsiella pneumoniae* and 9.35% for *Staphylococcus aureus*) and Yenehun Worku et al.^14^ in Ethiopia (63.6% for *Escherichia coli* and 13.7% for *Klebsiella pneumoniae*).The predominance of Enterobacteriaceae, especially *Escherichia coli*, is not surprising since itis the most frequent flora of the gastrointestinal tract from where it ascends to the urinary tract and uses its well‐characterized virulence factors to colonize the urinary tract.^29^, ^30^

Our results are in line with the data in the literature concerning the most frequent bacterial uropathogen during UTI in diabetics. In Madagascar, these are therefore the bacteria to be considered for probabilistic treatment. In our study, we found an excellent sensitivity rate of Enterobacteriaceae to Carbapenems, Nitrofurantoin, Amikacin, Fosfomycin and Ceftriaxone. This corroborates with the results of most other authors for the first four. Indeed, the study conducted by Jagadeeswaran et al found that *Escherichia coli* were 96% sensitive to Amikacin and to imipenem respectively and 81% to nitrofurantoin. In addition, *Klebsiella pneumoniae* was 81% sensitive to amikacin and 83% to imipenem.^31^ Similarly, Malik et al.^32^ found a sensitivity rate of 98% to nitrofurantoin and 98.9% to imipenem for *Escherichia coli* and Bagiret al.^16^ found a sensitivity rate of 87% for nitrofurantoin. Norafika et al. reported 93% sensitivity of *Escherichia coli* to fosfomycin.^13^ Among the possible explanations for this, the low use of these molecules in ambulatory care could be advanced. Amikacin, particularly, is not routinely used in our country, even in cases of allergy to third‐generation cephalosporin or quinolone and in cases of severe ANP, although its use is recommended in the guidelines.^3^ In addition, its risk of cochleo‐vestibular and renal toxicity, especially in diabetics whose renal function is already threatened or even failing for the most part, as well as its high cost and low accessibility might explain the limitation of its use.

The sensitivity to nitrofurantoin is also high in our country as elsewhere. In our country, its accessibility is very limited. Even if it could be proposed as a probabilistic antibiotherapy due to its efficacy, this molecule should be avoided in case of renal failure because its metabolites tend to accumulate and cause peripheral neuropathy. If there is no other choice, in case of renal failure, a dosage adjustment is necessary.^33^ Furthermore, its low serum concentration does not allow its use in parenchymal infections.^3^ It is a possibility of choice for treating simple acute cystitis and/or cystitis at risk of complication in Madagascar, given its sensitivity profile in our study.

Similarly, the sensitivity of Enterobacteriaceae to carbapenems, including imipenem, is excellent here like in India.^34^ The hypotheses are that these molecules are not widely available, very expensive and not widely prescribed. They should not be used as probabilistic antibiotherapy in order to preserve them as much as possible. They represent the reference therapeutic class for the treatment of infections by ESBLE.^31^ Fosfomycin still had an excellent place. This allows us to use it, as recommended, in the probabilistic antibiotherapy of cystitis.^3^ The fairly high sensitivity to ceftriaxone is, however, in disagreement with the results of many other authors such as Norafika et al (13%)^13^ and Jagadeeswaran et al (22%).^31^

This sensitivity is also increased compared to that reported by Dodo et al (51.61%).^30^ One hypothesis is that this molecule, which can only be administered by injection, is less used in self‐medication or is less prescribed in ambulatory care when patients consult private physicians before being hospitalized.

In our opinion, the use of injectable third generation cephalosporin, including Ceftriaxone, recommended as first‐line treatment for simple ANP, would retain its legitimacy. Enterobacteriaceae showed high resistance to more common antibiotics such as Amoxicillin, Sulfamethoxazole‐Trimethoprim and Amoxicillin‐Clavulanic Acid, which is in agreement with data from other studies.^13^, ^31^ We hypothesize that these molecules are widely used for other infections such as respiratory infections. In addition, their oral administration would favor their use in self‐medication. This might explain the high rate of resistance of Enterobacteriaceae to these molecules. Therefore, as recommended by the French Infectious Diseases Society, we refrain from using these molecules in the probabilistic antibiotherapy of ANP.^3^

The resistance of *Escherichia coli* to ciprofloxacin (39.02%) has increased, compared to that reported by Dodo et al. in the Nephrology Department Befelatanana Hospital (25.8%).This difference would be due to the inclusion criteria because theirs were interested in all subjects with UTI, whether diabetic or not.^30^ However, the frequency of fluoroquinolone resistance in our study was compatible with that reported in Indonesia (22%–67%).^13^ In our opinion, the recommendation to prescribe fluoroquinolone, including ciprofloxacin, is still appropriate in cases of ANP and prostatitis in our country, due to its wide availability, the possibility of its oral administration and its wide prostatic tissue diffusion, provided that the rules of prescription are strictly respected. This prescription should not be repeated within 6 months in order to avoid the appearance of fluoroquinolone‐resistant Enterobacteriaceae.^3^ A well‐conducted interrogation and a meticulous examination of the health records and medical prescriptions of the last 6 months, or even more, would therefore reduce this risk.

GPC in our study had shown a high sensitivity to most antibiotics except for Penicillin G. This is in agreement with the results obtained in Ethiopia.^14^, ^35^

The frequency of MDR identified in our study (21.31%) was comparable to that reported by Hamdan et al.^6^ in Sudan (28.2%) but was much lower than that reported by Yenehun Worku et al.^14^ in Ethiopia (100%). The variations in MDR frequencies found in these different studies could be explained in part by the difference of the inclusion criteria. Another possible explanation for these discrepancies is the nonuniformity of the antibiogram discs used in these different studies.

The two bacterial uropathogens that showed multidrug resistance in our series were *Escherichia coli* (27.91%) and Klebsiellapneumoniae (25%). Also in Indonesia, these two uropathogens were the most frequently MDR.^13^ Gomila et al. also reported the same trend in their multicenter study in Southern and Western Europe, Turkey, and Israel.^36^ This was not surprising since it has been shown for some time that the frequency of multidrug‐resistant *Escherichia coli* is increasing in diabetics and nondiabetics.^37^, ^38^ Niranjan et al claim that diabetes per se is a risk factor for infection by MDR *Esherichia coli*.‐^34^ *Klebsiella pneumoniae* is one of the main bacteria responsible for nosocomial infections, and the acquisition of multidrug resistance is mainly due to genetic changes in response to antibiotherapy.^39^ The lack of hygiene, the lack of health education, the high frequency of self‐medication, the existence of illicit sale of drugs, the existence of fake drugs and inferior quality or outdated drugs in circulation, the frequent use of broad‐spectrum antibiotics for antibioprophylaxis, and the lack of laboratory tests for the identification of pathogens would be at the root of this increase in the frequency of MDR,^14^, ^40^ especially Enterobacteriaceae.

Neither the male gender nor the age superior or equal to 65 years nor the notion of recent antibiotic use were found in our study as risk factors for infection by MDR, contrary to the results of the studies of Gomila et al.^36^ and Wright et al.^41^ This discrepancy could be explained by the fact that the studies of these other authors were interested in all UTI regardless of the terrain (diabetic or not). The sizes of the samples studied were also very different: 57 patients for our study, 435 for Wright et al.^41^ and 948 for Gomila et al.^36^ The patients should have presented a complicated UTI and therefore probably had a longer and cumulative pre‐hospital antibiotic therapy generating the selection of endogenous multi‐resistant flora.^38^ Moreover, our patients could have forgotten to bring their medical prescription at the time of hospitalization, although they could have benefited from antibiotic therapy beforehand.

Our result was nevertheless consistent with previous studies showing that Type 2 diabetics are more prone to UTI by MDR.^42^, ^43^ One of our hypotheses is that Type 2 diabetics, who are generally older than Type 1 diabetics, would have more infectious complications and would consume more antibiotics, which would promote MDR bacteria.

Poor glycemic control was also not associated in our study with infection by MDR. This is in agreement with the results reported by Bagir et al.^16^ Indeed, the mechanisms ofbacterial resistance to antibiotics including impermeability, enzymatic inactivation, changes in the target of the antibiotic and active efflux do not depend on carbohydratemetabolism.^44^

### 4.1 Limitations of the study

Our study has some limitations. Indeed, first, the urinary culture were performed in different laboratories, not guaranteeing the uniformity of the antibiogram discs used for the tests. Second, few patients were able to benefit from an HbA1c test since this test is not available in our institution and all paraclinical tests are not free of charge, forcing us to limit the number of biological tests to be requested.

## 5 CONCLUSION

In conclusion, in hospitals in Antananarivo, the epidemiological‐clinical and biological characteristics of urinary tract infection in our diabetic patients are similar to those reported in the literature. We found that the patients were predominantly female, the average age was around 60, the main clinical form of urinary tract infection was acute pyelonephritis and *Escherichia coli* was the main predominant bacterial uropathogen. More than one urinary culture out of five had isolated multidrug resistant bacteria, all extended‐spectrum beta‐lactamase‐producing Enterobacteriaceae. Only Type 2 diabetes appeared to be a predictive factor for infection by MDR in this study.

Despite this, Ceftriaxone, widely available, can be offered as probabilistic antibiotic therapy for parenchymal infection, if urine culture and antibiogram cannot be performed for some reason.

Nevertheless, prevention of UTI is always ideal and early consultation would make therapeutic management less costly and improve prognosis.

## AUTHOR CONTRIBUTIONS

**Rija Eric Raherison:** Conceptualization; data curation; formal analysis; investigation; methodology; software; visualization; writing – original draft; writing – review and editing. **Sitraka Angelo Raharinavalona:** Visualization; writing – review and editing. **Thierry Razanamparany:** Visualization; writing – review and editing. **Theo Njakasoa Randrianotahiana:** Conceptualization; data curation; formal analysis; investigation; methodology; software; writing – original draft. **Tsikinirina Valisoa Randrianomanana:** Visualization. **Miora Maeva Arielle Andrianiaina:** Visualization. **Andrianirina Dave Patrick Rakotomalala:** Supervision; validation; writing – review and editing. **Radonirina Lazasoa Andrianasolo:** Supervision; validation; writing – review and editing.

## FUNDING INFORMATION

The current study was not supported by any funding source.

## CONFLICT OF INTEREST STATEMENT

The authors have declared that they have no competing interest.

## ETHICS STATEMENT

This study was approved by the hierarchical heads and Review Board of the Joseph Raseta Befelatanana University Hospital Center, Antananarivo, Madagascar. Cf Hospital manager approbation letter on September 02, 2022.

## CONSENT

Written informed consent was obtained from the patient to publish this report.

## Data Availability

The data sets used and/or analyzed during the current study are available from the corresponding author on reasonable request.
